# Super-resolution triple-resonance NMR spectroscopy for the sequential assignment of proteins

**DOI:** 10.1126/sciadv.adv6246

**Published:** 2025-08-15

**Authors:** Olivia Gampp, Luca Wenchel, Peter Güntert, Piotr Klukowski, Roland Riek

**Affiliations:** ^1^ Institute of Molecular Physical Science, ETH Zürich, Vladimir-Prelog-Weg 2, CH-8093 Zürich, Switzerland.; ^2^ Institute of Biophysical Chemistry, Center for Biomolecular Magnetic Resonance, Goethe University Frankfurt am Main, 60438 Frankfurt am Main, Germany.; ^3^ Department of Chemistry, Tokyo Metropolitan University, Hachioji, 192-0397 Tokyo, Japan.

## Abstract

To study the structure and dynamics of proteins by nuclear magnetic resonance (NMR), sequence-specific assignment is needed, which can be obtained by acquiring and analyzing multiple triple-resonance experiments with the three-dimensional TROSY-HNCA, the most sensitive stand-alone experiment with which sequential assignment is, in principle, possible. However, gaining an unambiguous assignment solely from this spectrum is generally not possible because amino acid–type information cannot be gleaned only from the ^13^C^α^ shifts and the low resolution in the ^13^C dimension, which is limited by the homonuclear coupling of the ^13^C^α^ and ^13^C^β^ nuclei. Here, super-resolution NMR is applied to the TROSY-HNCA and HNcoCA experiments, yielding pseudo-decoupling, which results in a four- to fivefold resolution enhancement in the ^13^C dimension, essential for the assignment, which allows for straightforward assignment of proteins as large as 500 residues based on simulations.

## Teaser

Super-resolution triple-resonance NMR spectroscopy facilitates automated protein assignment.

## INTRODUCTION

Nuclear magnetic resonance (NMR) spectroscopy is a well-established and versatile multiprobe tool to study protein structure and dynamics related to function under physiological conditions in vitro and in cells (*1*, *2*). Assigning the peaks to their respective atoms in the protein is an essential step that can be achieved by acquiring suitable complementary triple-resonance experiments of a ^13^C,^15^N-labeled protein (*3*–*5*). A plethora of adaptations of these experiments exist for specific protein systems. For example, large proteins are additionally deuterated, and their spectra are acquired using the transverse relaxation-optimized spectroscopy [TROSY (*6*, *7*)] concept. For intrinsically disordered proteins, four- and higher-dimensional as well as projected experiments can be acquired. Methods that allow for fast measurements on well-behaved systems include nonuniform sampling (NUS) (*8*, *9*), CODED spectroscopy (*10*), and spin-state selective off-resonance decoupling (SITAR) (*11*). Additionally, various labeling strategies have been developed (*12*–*14*). Recently, the software package ARTINA not only established a fully automated procedure for chemical shift assignment and structure determination (*15*) but also shows that only a limited number of established two-dimensional (2D) and 3D NMR spectra are needed for the automated backbone assignment of medium-size proteins (up to a molecular weight of 20 kDa) (*15*, *16*). The total measurement time for these spectra amounts to ~1 week (*15*, *16*).

With the advent of ARTINA, novel triple-resonance NMR experiments and combinations thereof can be developed and acquired, which are adapted to the software. Here the super-resolution (SR) 3D HNCA is presented that enhances the resolution in the crucial assignment dimension by almost a factor of 5. Simulations with ARTINA indicate that, depending on the investigated protein system, a single 3D triple-resonance experiment is sufficient to get an automated assignment of the backbone within 24 hours for (^2^H), ^13^C,^15^N-labeled proteins.

## RESULTS

### Homodecoupling by dynamic weighting of the number of scans per increment in 2D [^13^C,^1^H]-HSQC

**Fig. 1.**
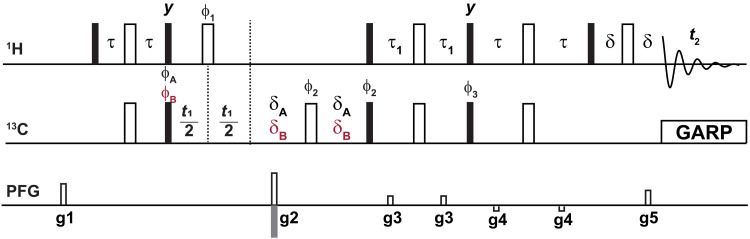
Modified pulse sequence of the sensitivity enhanced [^1^H,^13^C]-HSQC. The modified pulse sequence of the sensitivity enhanced [^1^H,^13^C]-HSQC (without ^13^CO decoupling) starting from the Bruker sequence hsqcetgpsi2. Narrow filled and wide unfilled bars represent 90° and 180° pulses, respectively. All pulses, unless otherwise specified, are applied along + x . Phase cycling is performed with $\phi_{1}=(x,x,-x,-x)$ , $\phi_{A}=\left(x,-x\right)$ , $\phi_{B}=\left(-x,x\right)$ , $\phi_{2}=\left(x,x,-x,-x\right)$ , $\phi_{3}=\left(y,y,-y,-y\right)$ , and $\phi_{\text{rec}}=\left(x,-x,-x,x\right)$ . $\phi_{A}$ is applied if $t_{1}<$ 11.1 ms − 2 ms, otherwise $\phi_{B}$ is used. The delays were chosen as τ = 1.7 ms and τ_1_ = 0.86 ms, δ is the time used for the gradient pulse and the recovery thereof; if *t*_1_ < 11.1 ms − 2 ms, then $\delta_{A}$ = 1 ms, otherwise $\delta_{B}$ = 3 ms (assuming ^1^*J*_CC_ = 38 Hz). Gradients 1 (g1) and 3 (g3) were applied for 600 μs, and the rest were 1 ms long. The gradient amplitudes were the following: g1 = 55%, g2 = 80%, g3 = 11%, g4 = −5%, and g5 = 20.1% of the maximum gradient strength. The sign of g2 is alternated within each increment to provide echo-antiecho selection. Globally optimized alternating phase rectangular pulses (GARP) was used to decouple the carbons during the acquisition. PFG, pulsed field gradient.

**Fig. 2.**
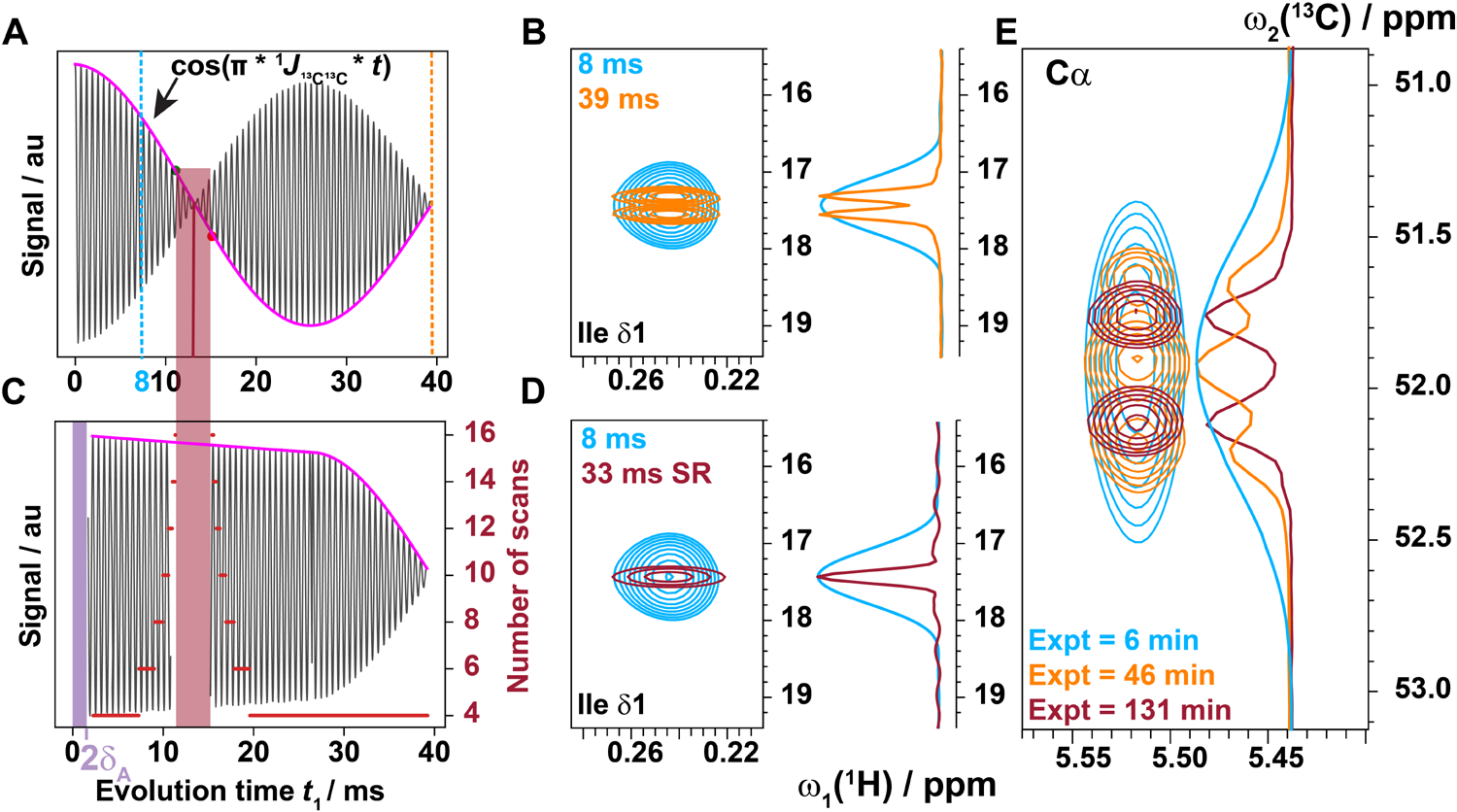
Overview of the SR concept ^13^C-decoupling applied to a [^13^C,^1^H]-HSQC. (**A**) Schematic of the free induction decay modulated by the ^1^*J*_CC_ coupling. (**B**) Close-up of an Ile δ peak in overlaid conventional [^13^C,^1^H]-HSQCs acquired with *t*_1max_ = 8 ms (cyan) or 42 ms (orange) in the ^13^C dimension. The ^1^*J*_CγCδ_ coupling causes the splitting reducing the resolution as can be seen in the cross section. (**C**) Schematic of the signal recorded with SR that has been pseudo-decoupled by dynamically increasing the number of scans to get rid of the decay of the cosine. The assumed coupling size optimized for methyl ^1^H-^13^C moieties was ^1^*J*_CC_ = 38 Hz. The *t*_1_ evolution times that are 2 ms on either side of the zero crossing are skipped (region shown in burgundy). The number of scans is not increased during the natural decay of the cosine after the zero crossing to allow for the signal to decay naturally toward 0. (**D**) The same Ile δ peak as in (C), acquired conventionally with *t*_1max_ = 8 ms compared to the SR [^13^C,^1^H]-HSQC with 33-ms acquisition (red). (**E**) The multiplet structure of all carbons with multiple neighboring ^13^C nuclei will be reduced by one coupling. Here, a ^13^C^α^ is decoupled from its C^β^, and the multiplet becomes a doublet because the ^1^*J*_CαC′_ coupling is still active. Color code: standard [^13^C,^1^H]-HSQC spectrum with *t*_1max_ = 8 ms (cyan) or 42 ms (orange) and SR (burgundy). The total experimental time (Expt) is given accordingly. au, arbitrary units.

In the original SR NMR method, the number of scans per increment in the indirect dimension is dynamically adjusted to counteract transverse relaxation, yielding cross peaks with reduced linewidth (*17*, *18*). Here, the same concept is applied to decouple the spectrum as demonstrated for the ^13^C-^1^H methyl moieties of a ^15^N,^13^C-labeled protein in the (nonconstant time) heteronuclear single-quantum correlation [^13^C,^1^H]-HSQC experiment by homodecoupling the 35- to 40-Hz ^1^*J*_CC_ coupling. Figure 1 shows the pulse sequence of a standard sensitivity-enhanced gradient-selective [^13^C,^1^H]-HSQC experiment. Its recording on the ^13^C,^15^N-labeled protein GB3 with an unusually long maximal evolution time *t*_1max_ of 39 ms in the ^13^C dimension yields a doublet of the cross peaks of ^13^C-^1^H methyl moieties caused by the *^1^J*_CC_ coupling (Fig. 2, A and B). To pseudo-homodecouple the cross peaks, the number of scans at a given time increment *t*_1_ is weighted by the integer number closest to 1/cos[π ^1^*J*_CC_ (*t*_1_ + 2δ_A_)] = 1/cos[π 38 Hz (*t*_1_ + 2δ_A_)], where 2δ_A_ is the time required for the gradient selection pulse sequence element shown in Fig. 1, because the ^1^*J*_CC_ coupling is active during both the latter element and the evolution time *t*_1_. In the pulse sequence setting proposed, δ_A_ = 1 ms. There is an additional point to be considered: At the zero crossing of the cosine at *t*_1_ + 2δ_A_ = 1/(2 ^1^*J*_CC_) = 13.1 ms (with ^1^*J*_CC_ = 38 Hz), the number of scans would become infinite, and unfeasible amounts of scans would be required nearby as shown in Fig. 2A. To circumvent this issue at an evolution time of *t*_1_ = 13.1 ms − 2δ_A_ − 2 ms = 9.1 ms and longer, δ_A_ is replaced by δ_B_ = δ_A_ + 2 ms as shown in Fig. 1. Along with it, the phase ϕ_A_ is altered to ϕ_B_ = ϕ_A_ + π to neutralize the sign change caused by the coupling. Accordingly, the number of scans is scaled according to the function 1/cos[π ^1^*J*_CC_ (*t*_1_ + 2δ_A/B_)] as demonstrated in Fig. 2C. With these settings, the spectrum with *t*_1max_ = 33 ms of ^13^C,^15^N-labeled GB3 is shown in Fig. 2 (D and E), as well as in fig. S3. The lack of the doublet along with a factor of 4 (i.e., 33/8) higher resolution than in a conventional HSQC is evident because of the longer acquisition time (i.e., 33 ms versus 8 ms). Artifacts that may occur include (i) linewidth distortion due to the digital number of scan sampling that can be rescued by a correction function as described elsewhere (*18*–*20*), and (ii) if a ^1^H-^13^C moiety has two neighboring ^13^C nuclei, then the multiplet will only be reduced to a doublet as shown in Fig. 2E. It is evident that this homonuclear-decoupling scheme can be applied to the HNCA experiment as we shall describe next.

### The SR HNCA experiment

**Fig. 3.**
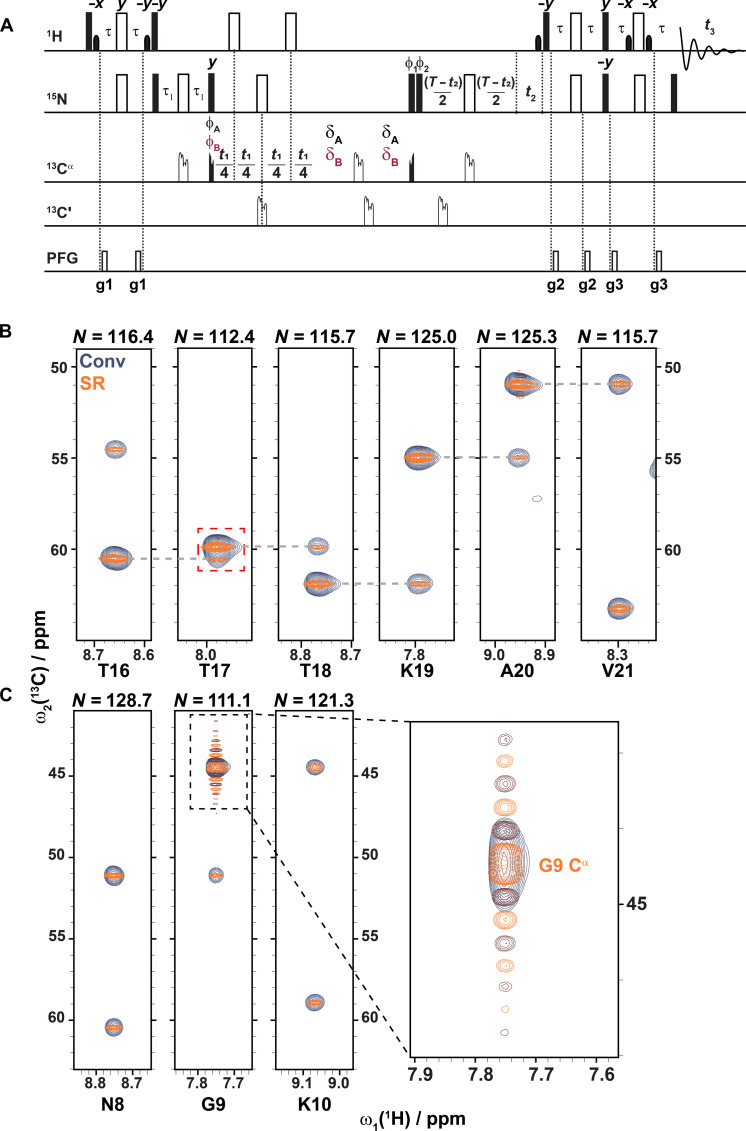
Backbone assignment with SR TROSY-HNCA. (**A**) Modified SR TROSY-HNCA pulse program starting from the Bruker sequence trhncagp3d2. The quadruple unfilled and filled bars are ^13^C Q3_surbop.1 and Q5_sebop.1 / Q5tr_sebop.1 pulses with pulse lengths of 231 and 300 μs, respectively. Black ones are applied on ^13^C^α^ at 53.2 ppm, and the gray ones are applied to^13^CO at 174 ppm. Phase cycling is performed with $\phi_{A}=\left(x,x,x,x\right)$ , $\phi_{B}=\left(-x,-x,-x,-x\right)$ , $\phi_{1}=\left(-x,x,y,-y\right)$ , $\phi_{2}=\left(-x,x,-y,y\right)$ , and $\phi_{\text{rec}}=\left(y,-y,-x,x\right)$ . $\phi_{A}$ is applied if *t*_1_ < 14.3 ms − 2 ms (assuming ^1^*J*_CαCβ_ = 35 Hz), otherwise $\phi_{B}$ is applied. The delays were chosen as τ = 2.3 ms, τ_1_ = 12 ms, and *T* = 24 ms; if *t*_1_ < 14.3 ms − 2 ms, then $\delta_{A}$ = 10 μs, otherwise $\delta_{B}$ = 2 ms. All gradients were applied for 1 ms with amplitudes g1 = 30%, g2 = 18%, and g3 = 44% of maximum gradient strength. SR was applied to pseudo-decouple ^1^H-^13^C^α^ moieties from ^13^C^β^ by dynamically adjusting the number of scans as shown in Fig. 2C. The assumed coupling size that gives the optimal spectra quality was *^1^J*_13C13C_ = 35 Hz. PFG, pulsed field gradient. (**B**) Six sequentially assigned strip plots from the SR TROSY-HNCA (orange/burgundy) overlaid on the conventionally acquired TROSY-HNCA (blue). The SR TROSY-HNCA is able to resolve T17 C^α^ from T16 C^α^ in the T17 strip marked in the red dashed box. (**C**) Three sequentially assigned strips. The glycine C^α^ shows a doublet with strong sinc wiggles while it appeared as a single broad peak in the conventionally acquired spectra.

With the goal to streamline the sequential assignment process to a single triple-resonance experiment, SR-based pseudo-homonuclear decoupling and linewidth reduction is applied to the 3D TROSY-HNCA experiment starting with the Bruker pulse sequence trhncagp3d2 using constant-time evolution in the ^15^N-dimension for ^13^C,^15^N-labeled proteins. (For ^2^H,^13^C,^15^N-labeled proteins, ^2^H decoupling during the ^13^C^α^ evolution time *t*_1_ needs to be added.) The pulse sequence is shown in Fig. 3A that includes, as in the case of the [^13^C,^1^H]-HSQC, δ_A_ = 10 μs and its replacement by δ_B_ = 2 ms at *t*_1_ = 14.3 ms − δ_B_ ms (with ^1^*J*_CαCβ_ = 35 Hz) and longer evolution times along with the replacement of ϕ_A_ by ϕ_B_ = ϕ_A_ + π to switch the sign due to the active ^1^*J*_CαCβ_ coupling (with 35 Hz). The number of scans is scaled by 1/cos(π ^1^*J*_CαCβ_ *t*_1_) for *t*_1_ < 12.3 ms and 1/cos[π ^1^*J*_CαCβ_ (*t*_1_ + 2δ_B_)] for *t*_1_ ≥ 12.3 ms. Otherwise, the 3D SR-TROSY-HNCA experiment follows the TROSY-HNCA pulse sequence with a magnetization transfer from ^1^H_N_ to ^15^N to ^13^C^α^ (*t*_1_ evolution) to ^15^N (*t*_2_ evolution) to ^1^H_N_ (acquisition), yielding per ^1^H-^15^N moiety one intraresidual cross peak with its ^13^C^α^ and one sequential cross peak with the N-terminally neighboring ^13^C^α^ as shown in Fig. 2B for a stretch of 6 residues. The almost five (i.e., 38 ms/8 ms) times higher resolution than in the corresponding 3D spectrum obtained with the standard pulse sequence using a maximal evolution time *t*_t,max_ of 8 ms dictated by the ^1^*J*_CαCβ_ coupling is evident and allows for the assignment of three sequential threonines that would normally require additional 3D spectra to lift the ambiguity. The price for resolution enhancement comes with a less than half loss in sensitivity as shown in fig. S1. The average number of scans required in the SR version is 4.86 compared to 4. The total acquisition time of the SR-HNCA was 16.25 hours compared to the 3-hour-long conventional acquired spectrum, and they had an average number of 4.86 and 4 scans, respectively, per increment. Additionally shown in fig. S1 is the modified pulse program acquired with a constant number of scans and by multiplying the window function 1/cos(π*35 Hz* *t*). However, you lose double the sensitivity compared to using the SR method. Another stretch of three residues (Fig. 3C) containing a glycine shows that its cross peak is now a doublet with strong sinc wiggles. This is due to the change in the sign of the phase and the increased number of scans causing its signal not to decay to zero by the end of the 38-s evolution time. The same modifications were made to the 3D SR-TROSY-HNcoCA pulse program shown in fig. S2. If the signal to noise is sufficient to reduce the measurement time, then NUS schemes can be used (*8*, *9*).

### The impact of SR triple-resonance experiments on automated sequential assignment using ARTINA

The SR triple-resonance experiments provide an advantage in NMR spectra interpretation by reducing the signal width in the C^α^ dimension. To explore quantitatively how this effect supports a downstream task, such as chemical shift assignment, and to examine opportunities for reducing the measurement time and facilitating the study of large protein systems, computer experiments with two simulated sets of spectra and automated assignment using ARTINA (*15*) were performed. The first set included [^15^N,^1^H]-HSQC, HNCA, SR-HNCA (SR), HNcoCA, SR-HNcoCA, and CBCAcoNH spectra of 89 proteins from the publicly available ARTINA benchmark dataset (*21*). These spectra were simulated using chemical shifts from the Biological Magnetic Resonance Data Bank (BMRB) and represent proteins ranging from 3 to 20 kDa, sizes commonly studied with macromolecular NMR spectroscopy (*22*).

To establish the second dataset, 85 subsets of proteins were randomly selected from the first dataset (each containing typically one to four proteins), such that each subset comprised 200 and 500 residues in total. Subsequently, the structures in each subset were concatenated with 10-residue glycine linkers, yielding multidomain systems. This process required structure regularization with CYANA (*23*) to prevent steric clashes between concatenated domains. The resulting multidomain systems were then used as input for simulating [^15^N,^1^H]-HSQC, HNCA, SR-HNCA, SR-HNcoCA, HNcoCA, and CBCAcoNH spectra, as for the first dataset.

**Fig. 4.**
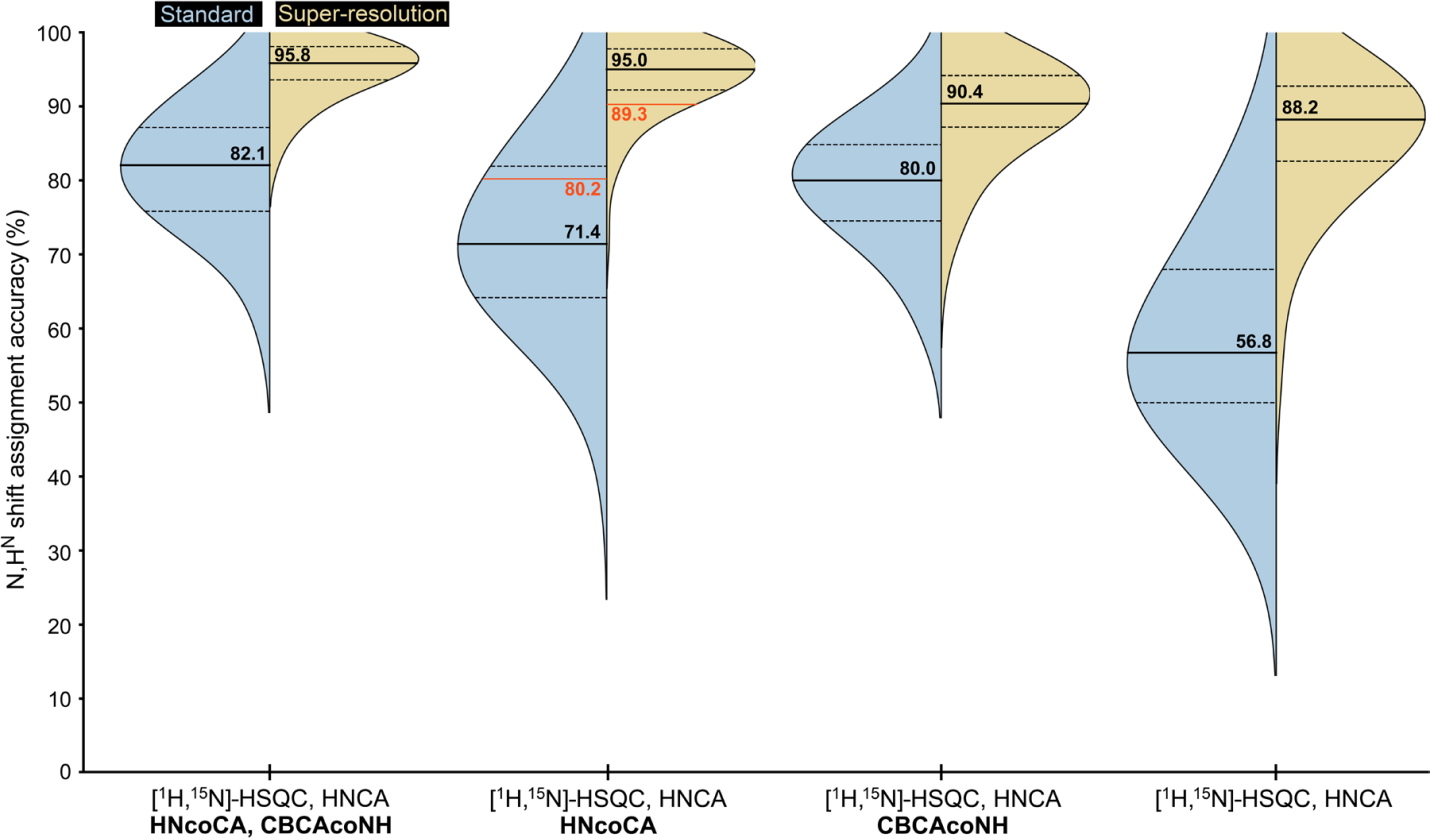
Accuracy of the automated chemical shift assignment (N,H^N^) evaluated for 89 proteins (dataset 1, 3 to 20 kDa) and four subsets of input spectra as labeled on the *x* axis. SR (results in yellow) was applied to the C^α^ dimension in the HNCA and HNcoCA experiments. Experimental data consisting of the [^1^H,^15^N]-HSQC, SR-HNCA, and SR-HNcoCA as well as the standard spectra were collected for the 20-kDa large protein Kras and automatically assigned with ARTINA as indicated by the red line and numbers.

In the first experiment (Fig. 4), the accuracy of the ARTINA-automated N,H^N^ shift assignment (*16*) of 89 proteins (dataset 1) with an AlphaFold2 structure prediction as input both with and without SR was compared, using four different sets of input spectrum types. The results consistently demonstrate the advantage of SR experiments across all tested settings, with median accuracy improvements ranging from 10.4% ([^15^N,^1^H]-HSQC, HNCA, and CBCAcoNH) to 31.4% ([^15^N,^1^H]-HSQC and HNCA). The improved accuracy originates from the reduced signal overlap in the SR setting, as well as increased cross-peak precision, which enhances the ability to accurately link C^α^s in HNCA experiments.

For 3- to 20-kDa proteins, the improvement obtained with SR varies depending on the amount of input data used. Intuitively, the overall benefit tends to decrease as the number of the spectra used for assignment increases, because the redundancy of HNCA, HNcoCA, and CBCAcoNH experiments compensates for signal overlap and peak picking ambiguity. In the SR setting (Fig. 4), the SR-HNcoCA experiment is more informative than CBCAcoNH (95% versus 90%), allowing assignment to be completed without measuring C^β^. Conversely, the effect is reversed when SR is not present (71% versus 80% accuracy).

**Fig. 5.**
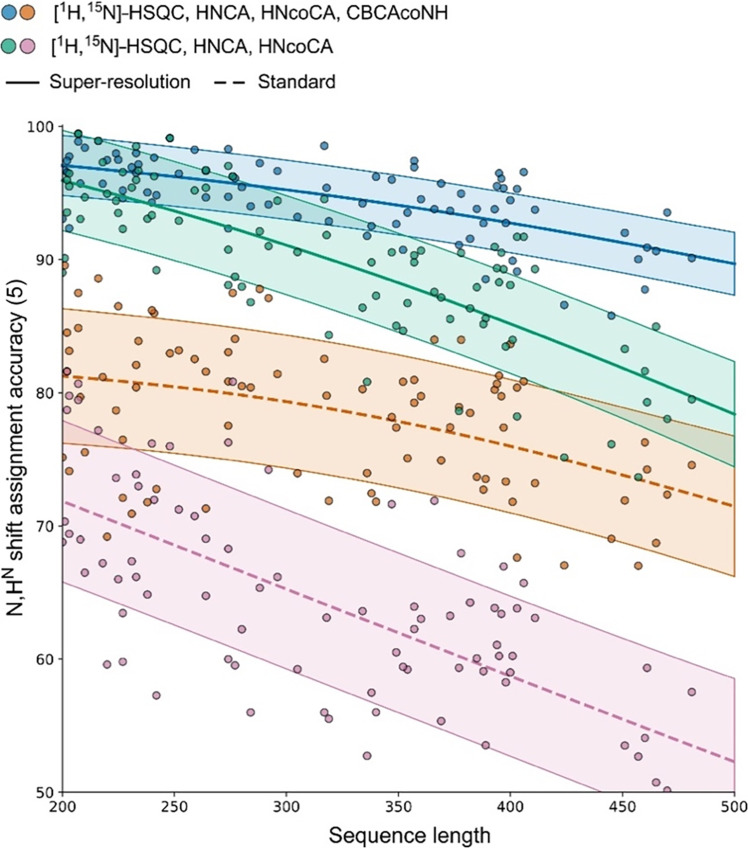
Accuracy of the automated chemical shift assignment (N,H^N^) evaluated for 85 multidomain proteins (dataset 2, 200 to 500 residues) and four subsets of input spectra. SR was applied to the C^α^ dimension in the HNCA and HNcoCA experiments. The solid trend line, a Gaussian process regression (GPR) fit, presents the performance of the SR approach both with and without C^β^ information from CBCAcoNH. The dotted trend lines show the results obtained with standard measurements. The highlighted area around the trend lines indicates the ±σ uncertainty in the GPR fit.

The ARTINA-automated N,H^N^ shift assignment was repeated using spectra simulated for 85 larger multidomain proteins with 200 to 500 residues (Fig. 5; dataset 2). The reported accuracies follow a consistent pattern, with SR experiments being beneficial in all tested settings, yielding a median improvement of 14.7 to 28.7% in assignment accuracy. When plotted against the sequence length, the assignment accuracy decreases as the system size increases. The regression curves plotted for the same input spectra with and without SR show a similar pattern with a vertical offset, highlighting the positive impact of the SR approach.

## DISCUSSION

The backbone chemical shift assignment of proteins using triple-resonance experiments of small- and medium-size ^13^C,^15^N-labeled proteins with a molecular weight up to 25 kDa and also large ^2^H,^13^C,^15^N-labeled proteins with a molecular weight up to ca 100 kDa is well established (*14*). It is based on a suite of NMR experiments including eventually specific labeling strategies (*24*). With this input, fully automated assignment procedures for small- and medium-size proteins are also available (*15*, *25*). In an attempt to streamline the automated assignment procedure using triple-resonance experiments, a SR TROSY-HNCA is proposed here. A factor of almost five times higher resolution along the ^13^C^α^ dimension is obtained. The impact on the automated chemical shift assignment using the ARTINA software is further demonstrated. This analysis shows that only one or two triple-resonance experiments would suffice to obtain the sequential backbone assignment of small- and medium-size proteins. This was experimentally verified by measuring the [^1^H,^15^N]-HSQC, SR-HNCA, and SR-HNcoCA as well as the conventional spectra of the ^13^C,^15^N-labeled 20-kDa large protein Kras. ARTINA was able to sequentially assign ~10% more of the backbone when using the SR spectra (89.3%) compared to 80.2% when using the conventional spectra. It further indicates that, for large systems with a molecular weight up to 50 kDa, fully automated sequential assignment may be possible. This may be important because the HNCACB/CBCAcoNH-type experiments, which are often critical for the sequential assignment of large systems, show low signal to noise.

The method presented here allows for the homodecoupling during the indirect ^13^C dimension by experimental means. Virtual homodecoupling along the ^13^C dimension by the software FID-Net (*26*) using a similar evolution time can also be obtained (fig. S4). The benefit of acquiring the spectra using the SR technique is that the peak positions, which are used for the sequential assignment, are consistent within a spectrum as they are experimentally defined while this might not be the case by the software manipulation of the spectra (fig. S4). In contrast, the SR spectrum must be recorded 1.3 times longer (20 hours compared to 15.5 hours).

As a side result of the presented SR 3D TROSY-HNCA experiment, a SR [^13^C,^1^H]-HSQC was studied. It comprises a spectrum that is fully decoupled for methyl resonances (with the exception of methionine that shows a doublet). As in the case of the SR TROSY-HNCA, a resolution enhancement of 4 to 5 is obtained when compared to a conventional HSQC experiment. The approach enables high-resolution methyl-based experiments without special labeling.

## MATERIALS AND METHODS

NMR experiments were acquired on a Bruker Avance III HD 700 MHz (16.4 T) equipped with a 5-mm cryogenically cooled proton-optimized ^1^H[^13^C/^15^N] TCI probe at 298 K with TopSpin version 4.1.1. Processing and visualization of all NMR spectra were done with TopSpin (Bruker) or PROSA (*27*). The reference and SR [^13^C,^1^H]-HSQC had 2048 × 2440 and 2048 × 2092 points, respectively, with a sweep width (SW) of 165 parts per million (ppm) (^13^C) and 13.0 ppm (^1^H) and a 0.6-s recycling delay. The reference and SR TROSY-HNCA were collected with 2048 × 40 × 440 and 2048 × 40 × 400, respectively, with an SW of 30 ppm (^13^C), 35 ppm (^15^N), and 17.9 ppm (^1^H) also with a recycling delay of 0.6. All spectra were processed using a squared cosine window function (SSB = 2) for both dimensions. Reference HSQC and HNCA spectra were processed twice, once with TDEFF of 2048 × 464 and 2048 × 84 to get the spectral resolution of 42- and 8-ms acquisition in the indirect dimension respectively. SR spectra were processed additionally in PROSA for smoothing in the indirect dimension as described by Gampp *et al*. (*20*), to correct for the discrepancy between the number of scans dictated by the function 1/cos(π ^1^*J*_CαCβ_ *t*_1_) and the integer number of scans that can be acquired. The protein sample used was an old sample of the uniformly ^15^N,^13^C-labeled GB3 [350 μl of 4 mM protein solution in 97% H_2_O, 3% D_2_O, 50 mM potassium phosphate buffer (pH 6.5), and sodium azide (0.5 mg/ml) measured at 298 K] (*28*).

The HNCA spectra and the respective artifacts and noise were simulated with in-house software (not published). All simulations were carried out using a *B*_0_-field corresponding to a ^1^H Larmor frequency of 700 MHz. The number of complex points in the time domain was 128 for ^15^N, 64 for ^13^C, and 512 for ^1^H for the regular spectra and 128 for ^15^N, 256 for ^13^C, and 512 for ^1^H for the SR spectra. The SWs were chosen such that all peaks lie within the spectral width. For the SR spectra, the scalar coupling interaction between ^13^C^α^ and ^13^C^β^ was neglected.

## Supplementary Materials

### This PDF file includes:

Figs. S1 to S4 HNCA Pulse Program Example vclist
