# Supplementary material for: Super-resolution triple-resonance NMR spectroscopy for the sequential assignment of proteins
Source: Sci Adv. 2025 Aug 15;11(33):eadv6246. doi: 10.1126/sciadv.adv6246 (PMC12356247; doi:10.1126/sciadv.adv6246)
Supplement: Supplementary file 1 — Figs. S1 to S4 HNCA Pulse Program Example vclist [file sciadv.adv6246_sm.pdf]

Science Advances
AAAS

Supplementary Materials for

Super-resolution triple-resonance NMR spectroscopy for the sequential assignment of proteins

Olivia Gampp *et al.*

Corresponding author: Roland Riek, roland.riek@phys.chem.ethz.ch

Sci. Adv. **11**, eadv6246 (2025)
DOI: 10.1126/sciadv.adv6246

This PDF file includes:

Figs. S1 to S4
HNCA Pulse Program
Example vclist

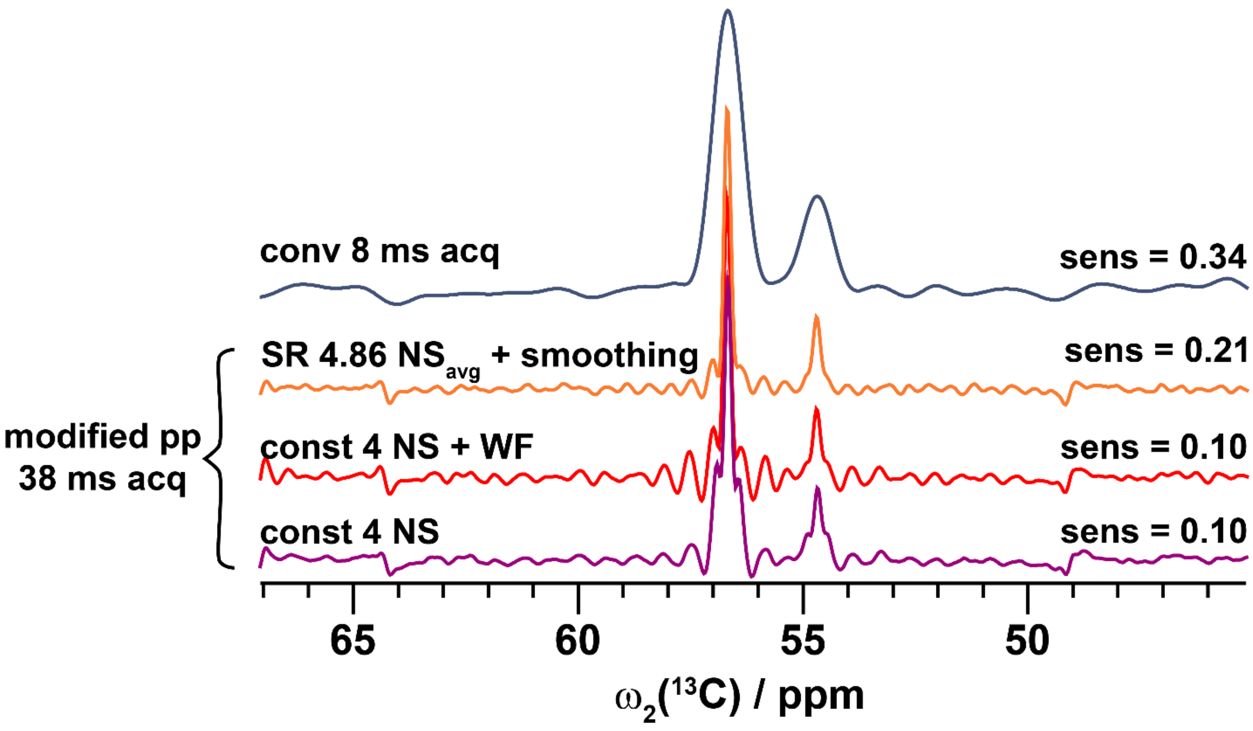

Fig. S1 Sensitivity of super-resolution acquisition in comparison to standard acquisition for a factor of 4-5 resolution enhancement. A cross section of a peak used to illustrate the sensitivity. The measured sensitivity indicated on the right (in arbitrary units) was calculated as the signal-to-noise ratio normalized by the square root of the measurement time of the individual experiments. For a gain of 4-5 in resolution in the SR acquired spectrum, less than half in sensitivity is lost compared to the conventionally acquired spectrum. The conventional acquired spectrum took 3 hours to acquire, using the modified pulse program and a constant number of scans just under 14 hours and when increasing the number of scans to counteract the J-coupling (SR) 16.25 hours. The sensitivity lost when keeping the scans constant cannot be substantially improved when processing with the window function (WF) = 1/cos(π*35 Hz* t).

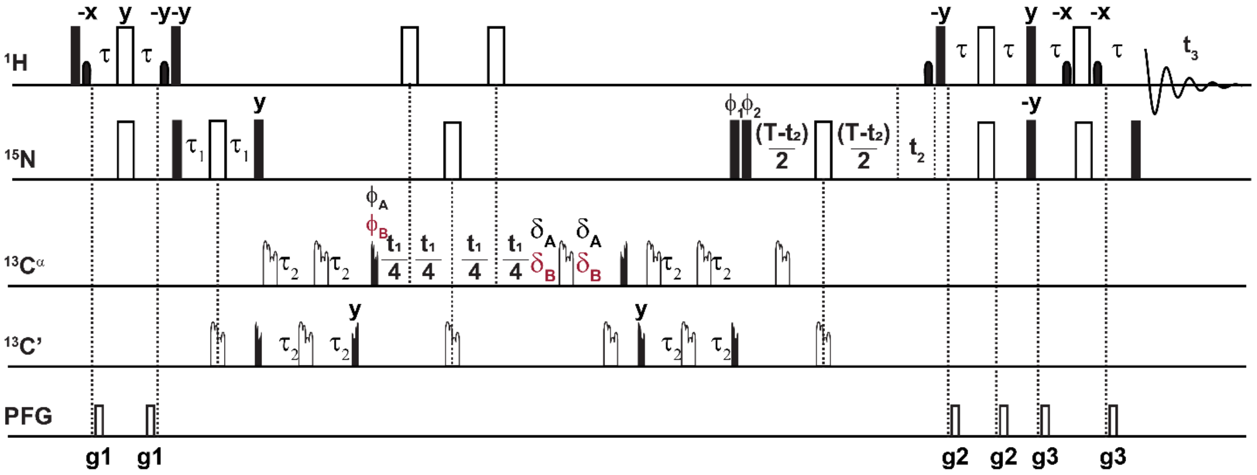

Fig. S2 Modified TROSY-HNcoCA pulse program.

Modified TROSY-HNcoCA pulse program starting from the Bruker sequence trhncocagp3d. The quadruple unfilled and filled bars are ^{13}C Q3_surbop.1 and Q5_sebop.1 / Q5tr_sebop.1 pulses with pulse lengths of 231 µs and 300 µs respectively. Phase cycling is performed with $\phi_A = (x, x,\ x,\ x)$, $\phi_B = (-x, -x, -x, -x)$, $\phi_1 = (-x, x,\ y, -y)$, $\phi_2 = (-x, x, -y,\ y)$, $\phi_{rec} =\ (y, -y, -x, x)$. ϕ_A is applied if $t_1 < 14.3$ ms $-$ 2 ms else ϕ_B. The delays were chosen as τ = 2.3 ms, τ_1 = 12 ms, τ_2 = 4 ms, T = 24 ms, if $t_1 < 14.3$ ms $-$ 2 ms than δ_A = 10 µs otherwise δ_B = 2 ms. All gradients were applied for 1 ms. The gradient amplitudes were the following: g1 = 30%, g2 = 18%, g3 = 44% of the maximum gradient strength.

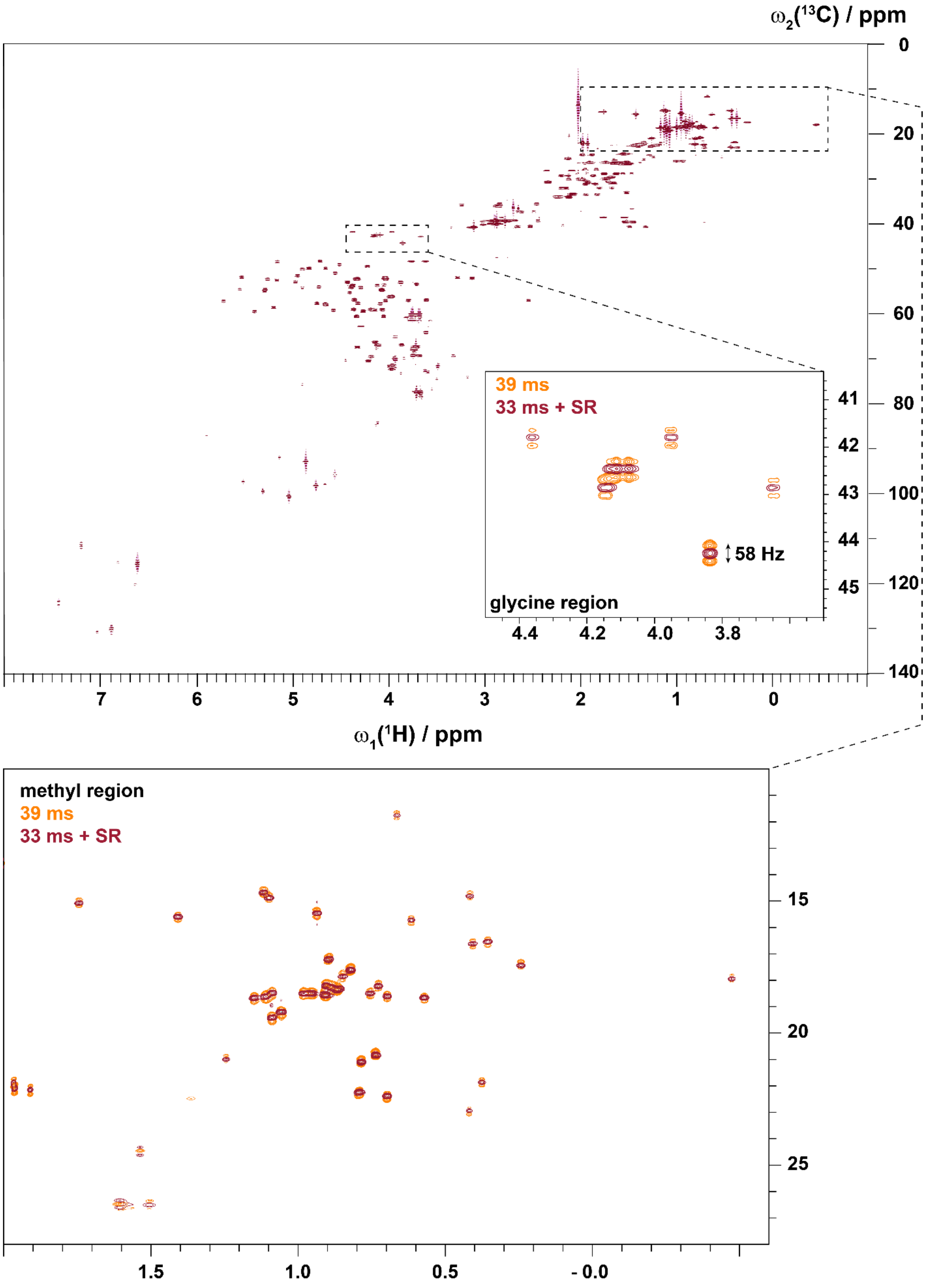

Fig. S3 (SR) [^{13}C, ^{1}H]-HSQC spectrum of GB3.

SR [^{13}C, ^{1}H]-HSQC spectrum of GB3. The glycine as well as the methyl region are zoomed in (burgundy) and overlayed with the conventional [^{13}C, ^{1}H]-HSQC (orange).

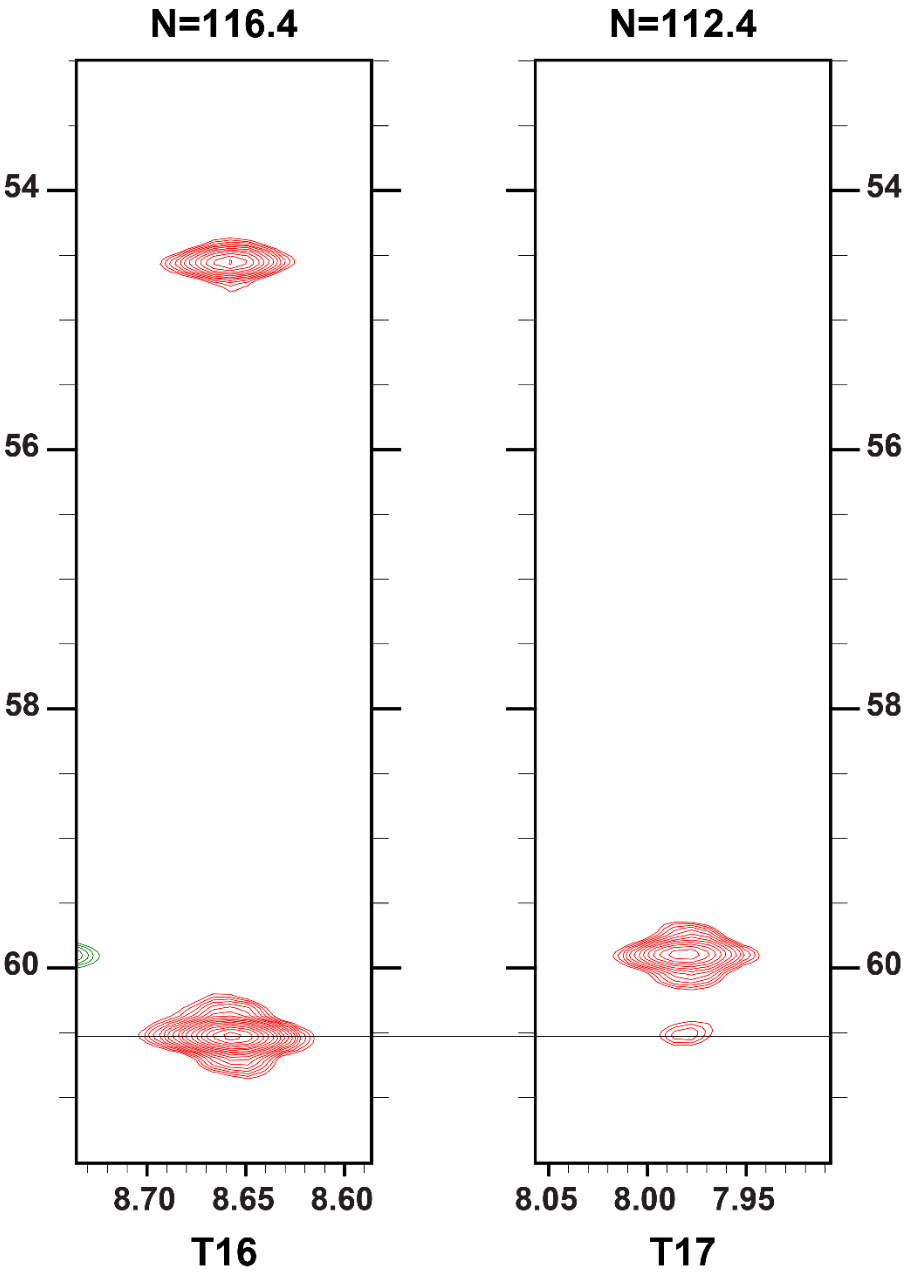

Fig. S4 Virtual ^{13}C homo decoupling by the software package FIDNET for the strips T16 and T17 measuring a TROSY-HNCA with 42 ms ^{13}C resolution

The cross peaks of the two strips of T16 and T17 are homo decoupled by FIDNET similar to the strips of the SR-TROSY HNCA shown in Figure 2B yielding a similar resolution. However, the chemical shifts of the T16 C^{α} varies slightly within the two strips of the FIDNET treated spectrum. This could cause uncertainty when sequentially assigning the backbone. It is mentioned that the super resolution spectrum is measured 1.3 times longer than acquiring the conventional spectrum needed for the virtual decoupling (20 h compared to 15.5 h).

```
;trhncagp3d2
;avance-version (15/02/27)
;TROSY-HNCA
;3D sequence with
;   inverse correlation for triple resonance
;      via TROSY and inept transfer steps
;
;      F1(H) -> F3(N) -> F2(Ca,t1) -> F3(N,t2) -> F1(H,t3)
;
;on/off resonance Ca and C=O pulses using shaped pulse
;phase sensitive (t1)
;phase sensitive using Echo/Antiecho (t2)
;using constant time in t2
;with H-1 180degree pulses in t1
;(use parameterset TRHNCAGP3D2)
;
;(M. Salzmann, K. Pervushin, G. Wider, H. Senn & K. Wuethrich, Proc.
;   Natl. Acad. Sci. USA 95, 13585-13590 (1998))
;A. Eletsky, A. Kienhoefer & K. Pervushin,
;   J. Biomol. NMR 20, 188-180 (2001)
;
;$CLASS=HighRes
;$DIM=3D
;$TYPE=
;$SUBTYPE=
;$COMMENT=


prosol relations=<triple>


#include <Avance.incl>
#include <Grad.incl>
#include <Delay.incl>
define list<loopcounter> SUPER=<$VCLIST>

"p2=p1*2"
"p22=p21*2"
"d11=30m"
"d12=20u"

"d23=14m"
"d26=2.3m"
"d17=10u"
```

```
"d0=3u"
"d10=3u"
"d30=d23-p11-8u-p1"


"in0=inf1/4"
"in10=inf2/2"

"in30=in10"

"td2=tdmax(td2,d30*2,in30)"


"DELTA=d0*4+larger(p14,p22)-p14+p2*2-4u"
"DELTA1=d26-p11-p16-d16-8u"
"DELTA2=d23-d10-p14-p21*4/3.1416"
"DELTA3=d26-p16-d16"

"l1=1"
"l0=1"
"l8=0"
"l3=td2/2"
"l10=td1/2"

"spoff2=0"
"spoff3=0"
"spoff5=bf2*(cnst21/1000000)-o2"
"spoff8=0"


;aqseq 321


1 d11 ze
  d11
;----Beginning of dummy scan loop section
2 d1 pl1:f1
  50u UNBLKGRAD

  p1 ph1
  4u pl0:f1
  (p11:sp1 ph3:r):f1
  4u
  p16:gp1
  d16 pl1:f1
  DELTA1
```

```
  (center (p2 ph2) (p22 ph1):f3 )
  DELTA1
  p16:gp1
  d16 pl0:f1
  (p11:sp1 ph4:r):f1
  4u
  4u pl1:f1
  (p1 ph4):f1

  (p21 ph1):f3
  d23
  (center (p14:sp3 ph1):f2 (p22 ph1):f3 )
  d23
  (p21 ph2):f3

  (p13:sp2 ph5):f2
  d0
  (p2 ph1):f1
  d0
  (center (p14:sp5 ph1):f2 (p22 ph1):f3 )
  d0
  (p2 ph1):f1
  d0
  (p14:sp3 ph1):f2
  DELTA
  (p14:sp5 ph1):f2
  4u
  (p13:sp8 ph1):f2
if "l0==1" goto 41
  (p21 ph6):f3
goto 42
41 (p21 ph7):f3

;;  if "l0 %2 == 1"
;;     {
;;     (p21 ph6):f3
;;     }
;;  else
;;     {
;;     (p21 ph7):f3
;;     }

42  d10
  (p14:sp5 ph1):f2
  DELTA2
  (center (p14:sp3 ph1):f2 (p22 ph1):f3 )
```

```
  d30 pl0:f1
  (p11:sp1 ph1:r):f1
  4u
  4u pl1:f1

  (p1 ph8)
  p16:gp2
  d16
  DELTA3
  (center (p2 ph1) (p22 ph1):f3 )
  DELTA3
  p16:gp2
  d16
  (center (p1 ph1) (p21 ph8):f3 )
  p16:gp3
  d16
  DELTA1 pl0:f1
  (p11:sp1 ph3:r):f1
  4u
  4u pl1:f1
  (center (p2 ph1) (p22 ph1):f3 )
  4u pl0:f1
  (p11:sp1 ph3:r):f1
  DELTA1
  p16:gp3
  d16 pl1:f1
  4u BLKGRAD
            ;10u iu0
lo to 2 times l18

;---End of dummy scan loop
3 100u
4 100u
5 100u
6 100u
7 100u
8 d1 pl1:f1
            "l7 = SUPER[l8]"
  50u UNBLKGRAD
  p1 ph1
  4u pl0:f1
  (p11:sp1 ph3:r):f1
  4u
  p16:gp1
  d16 pl1:f1
  DELTA1
```

```
  (center (p2 ph2) (p22 ph1):f3 )
  DELTA1
  p16:gp1
  d16 pl0:f1
  (p11:sp1 ph4:r):f1
  4u
  4u pl1:f1
  (p1 ph4):f1

  (p21 ph1):f3
  d23
  (center (p14:sp3 ph1):f2 (p22 ph1):f3 )
  d23
  (p21 ph2):f3

  ;(p13:sp2 ph5):f2
  if "d0 > d20*0.25"{
  (p13:sp2 ph15):f2
  } else {(p13:sp2 ph5):f2
  }
  d0
  (p2 ph1):f1
  d0
  (center (p14:sp5 ph1):f2 (p22 ph1):f3 )
  d0
  (p2 ph1):f1
  d0
if "d0 > d20*0.25"{
"d17 = d21"
}
  d17
  (p14:sp3 ph1):f2
  d17
  DELTA
  (p14:sp5 ph1):f2
  4u
  (p13:sp8 ph1):f2
if "l1==1" goto 31
  (p21 ph6):f3
goto 32
31 (p21 ph7):f3

;  if "l0 %2 == 1"
;     {
;     (p21 ph6):f3
;     }
```

```
;  else
;     {
;     (p21 ph7):f3
;     }

32  d10
  (p14:sp5 ph1):f2
  DELTA2
  (center (p14:sp3 ph1):f2 (p22 ph1):f3 )
  d30 pl0:f1
  (p11:sp1 ph1:r):f1
  4u
  4u pl1:f1

  (p1 ph8)
  p16:gp2
  d16
  DELTA3
  (center (p2 ph1) (p22 ph1):f3 )
  DELTA3
  p16:gp2
  d16
  (center (p1 ph1) (p21 ph8):f3 )
  p16:gp3
  d16
  DELTA1 pl0:f1
  (p11:sp1 ph3:r):f1
  4u
  4u pl1:f1
  (center (p2 ph1) (p22 ph1):f3 )
  4u pl0:f1
  (p11:sp1 ph3:r):f1
  DELTA1
  p16:gp3
  d16 pl1:f1
  4u BLKGRAD
  (p21 ph1):f3
  ;go=2 ph31
  ;d11 mc #0 to 2
  goscnp ph31
  3m ipp5 ipp15 ipp6 ipp7 ipp31
          lo to 3 times l7
          100u wr #0 if #0 zd
   ;  F1PH(calph(ph5, +90), caldel(d0, +in0))
   ; F2EA(calph(ph8, +180) & calclc(l0, 1), caldel(d10, +in10) & caldel(d30, -in30))
  30u ip8
```

```
  30u ip8
  30u iu1
  ;30u iu0
lo to 4 times 2
  ;30u rp8
  30u ru1
  ;30u ru0
  30u id10
  30u dd30
lo to 5 times l3
  30u rd10
  30u rd30
  30u ip5
  30u ip15
lo to 6 times 2
  30u id0
  30u iu8
lo to 7 times l10

exit


ph1=0
ph2=1
ph3=2
ph4=3
ph5=0 0 0 0 ;2 2 2 2
ph15=2 2 2 2
ph7=2 0 1 3
ph6=2 0 3 1
ph8=3
ph31=1 3 2 0; 3 1 0 2


;pl0 : 0W
;pl1 : f1 channel - power level for pulse (default)
;pl3 : f3 channel - power level for pulse (default)
;sp1: f1 channel - shaped pulse  90 degree  (H2O on resonance)
;sp2: f2 channel - shaped pulse  90 degree  (Ca on resonance)
;sp3: f2 channel - shaped pulse 180 degree  (Ca on resonance)
;sp5: f2 channel - shaped pulse 180 degree  (C=O off resonance)
;sp8: f2 channel - shaped pulse  90 degree  (Ca on resonance)
;                   for time reversed pulse
;p1 : f1 channel -  90 degree high power pulse
;p2 : f1 channel - 180 degree high power pulse
;p11: f1 channel -  90 degree shaped pulse             [1 msec]
```

```
;p13: f2 channel -  90 degree shaped pulse
;p14: f2 channel - 180 degree shaped pulse
;p16: homospoil/gradient pulse                       [1 msec]
;p21: f3 channel -  90 degree high power pulse
;p22: f3 channel - 180 degree high power pulse
;d0 : incremented delay (F1 in 3D)                   [3 usec]
;d1 : relaxation delay; 1-5 * T1
;d10: incremented delay (F2 in 3D)                   [3 usec]
;d11: delay for disk I/O                             [30 msec]
;d12: delay for power switching                      [20 usec]
;d16: delay for homospoil/gradient recovery
;d23: 1/(4J(NCa)                                     [12 msec]
;d26: 1/(4J'(NH)                                     [2.3 msec]
;d30: decremented delay (F2 in 3D) = d23-p11-8u-p1
;cnst21: CO chemical shift (offset, in ppm)
;cnst22: Calpha chemical shift (offset, in ppm)
;o2p: Calpha chemical shift (cnst22)
;inf1: 1/SW(Ca) = 2 * DW(Ca)
;inf2: 1/SW(N) = 2 * DW(N)
;in0: 1/(4 * SW(Ca)) =  (1/2) DW(Ca)
;nd0: 4
;in10: 1/(2 * SW(N)) = DW(N)
;nd10: 2
;in30: = in10
;ns: 4 * n
;ds: = 0
;td1: number of experiments in F1
;td2: number of experiments in F2       td2 max = 2 * d30 / in30
;FnMODE: States-TPPI (or TPPI) in F1
;FnMODE: echo-antiecho in F2


;use gradient ratio:    gp 1 : gp 2 : gp 3
;                         30 :   18 :   44

;for z-only gradients:
;gpz1: 30%
;gpz2: 18%
;gpz3: 44%

;use gradient files:
;gpnam1: SMSQ10.100
;gpnam2: SMSQ10.100
;gpnam3: SMSQ10.100
```

;$Id: trhncagp3d2,v 1.21 2017/05/04 17:00:59 ber Exp $

vclist (generated for: 200r increments, dwell time 189.3324 μs and $^{1}J_{C\alpha C\beta}$ = 35 Hz):
4
4
4
4
4
4
4
4
4
4
4
4
4
4
4
4
4
4
4
4
4
4
4
4
4
4
4
4
4
4
4
4
4
4
4
4
4
4
4
4
4
4
4
4
4

4
4
4
4
4
4
8
8
8
8
8
8
8
8
8
12
12
12
12
16
16
16
12
12
12
12
8
8
8
8
8
8
8
8
8
4
4
4
4
4
4
4
4
4
4
4

4
4
4
4
4
4
4
4
4
4
4
4
4
4
4
4
4
4
4
4
4
4
4
4
4
4
4
4
4
4
4
4
4
4
4
4
4
4
4
4
4
4
4
4
4
4

4
4
4
4
4
4
4
4
4
4
4
4
4
4
4
4
4
4
4
4
4
4
4
4
4
4
4
4
4
4
4
4
4
4
4
4
4
4
4
4
4
4
4
4
4
4

4
4
4
4
4
4
4
4
4
4
4
4
4
4
4
4
4
